# Comparative analysis of large language models in clinical diagnosis: performance evaluation across common and complex medical cases

**DOI:** 10.1093/jamiaopen/ooaf055

**Published:** 2025-06-12

**Authors:** Mehmed T Dinc, Ali E Bardak, Furkan Bahar, Craig Noronha

**Affiliations:** Department of Medicine, Boston Medical Center, Boston, MA 02118, United States; Department of Medicine, St Elizabeth’s Medical Center, Boston, MA 02135, United States; Department of Medicine, Mount Auburn Hospital, Harvard Medical School, Cambridge, MA 02138, United States; Department of Medicine, Boston Medical Center, Boston, MA 02118, United States

**Keywords:** large language models, clinical decision support, artificial intelligence, medical diagnosis

## Abstract

**Objectives:**

This study aimed to systematically evaluate and compare the diagnostic performance of leading large language models (LLMs) in common and complex clinical scenarios, assessing their potential for enhancing clinical reasoning and diagnostic accuracy in authentic clinical decision-making processes.

**Materials and Methods:**

Diagnostic capabilities of advanced LLMs (Anthropic’s Claude, OpenAI’s GPT variants, Google’s Gemini) were assessed using 60 common cases and 104 complex, real-world cases from Clinical Problem Solvers’ morning rounds. Clinical details were disclosed in stages, mirroring authentic clinical decision-making. Models were evaluated on primary and differential diagnosis accuracy at each stage.

**Results:**

Advanced LLMs showed high diagnostic accuracy (>90%) in common scenarios, with Claude 3.7 achieving perfect accuracy (100%) in certain conditions. In complex cases, Claude 3.7 achieved the highest accuracy (83.3%) at the final diagnostic stage, significantly outperforming smaller models. Smaller models notably performed well in common scenarios, matching the performance of larger models.

**Discussion:**

This study evaluated leading LLMs for diagnostic accuracy using staged information disclosure, mirroring real-world practice. Notably, Claude 3.7 Sonnet was the top performer. Employing a novel LLM-based evaluation method for large-scale analysis, the research highlights artificial intelligence’s (AI’s) potential to enhance diagnostics. It underscores the need for useful frameworks to translate accuracy into clinical impact and integrate AI into medical education.

**Conclusion:**

Leading LLMs show remarkable diagnostic accuracy in diverse clinical cases. To fully realize their potential for improving patient care, we must now focus on creating practical implementation frameworks and translational research to integrate these powerful AI tools into medicine.

## Introduction

### Background and significance

The use of artificial intelligence (AI) in medicine has seen a recent surge of interest, especially following the introduction of large language models (LLMs).^1–3^ While AI in medicine predates LLMs with rule-based systems,^4^^,^^5^ these earlier systems had limited accuracy and struggled with clinical complexity. In contrast, LLMs represent a clear shift forward. Their enhanced ability to understand natural language combined with exceptional skill in pattern recognition^6^ creates new opportunities for clinical integration.^7^ As such, LLMs hold great promise to significantly advance medical diagnostics.

Accurate and timely clinical diagnosis is essential, yet misdiagnosis remains a major health-care challenge, affecting around 1 in 9 patients and causing serious morbidity, mortality, and increased health-care costs.^8^ Large language models have powerful abilities in recognizing patterns and synthesizing information, making them a promising tool to improve diagnostic accuracy.^9^ However, current LLM evaluations often use simplified, static scenarios and few models.^10–14^ These methods do not adequately reflect the iterative and staged decision-making typical of real-world clinical practice. They also overlook the possibility that LLMs from different providers may offer better performance.

To address this gap, we comparatively analyzed leading LLMs from major AI providers, including recent models not previously assessed in clinical diagnosis. Our study uniquely simulates clinical practice by adopting a staged information disclosure approach, closely reflecting the iterative process clinicians use in real-world diagnostic workflows. To keep our evaluation clinically relevant yet challenging, we tested the model using complex, real-world scenarios taken from the Clinical Problem Solvers’ (CPSolvers)^15^ morning rounds. We also used common cases with subtle variations reflecting real-world deviations from textbook examples. Our goal was to assess whether LLMs could identify these subtle differences, a skill essential for clinicians.

Our hypothesis is 2-fold: (1) LLMs will exhibit significant performance variations, especially in complex cases requiring nuanced clinical reasoning and (2) some smaller LLMs can perform similarly to larger models in specific situations, suggesting potential for wider adoption that is also cost-effective.

## Materials and methods

### Case selection and data preparation

Our study evaluated LLM performance using 2 distinct case sets. The first set comprised 60 cases representing common clinical presentations. Case selection used a clinician consensus list (reflecting common Boston-area practice and frequent US ED presentations). Cases were intentionally designed with subtle deviations from classic textbook presentations to enhance diagnostic challenge and reflect real-world clinical variability. These cases were developed and peer-reviewed by 2 internal medicine residents on the study team to ensure realistic, nuanced scenarios.

The second set included 104 real patient cases sourced from the publicly available CPSolvers website, characterized by uncommon conditions or atypical presentations.

Cases were structured into progressive stages to closely mirror real-world clinical practice in internal medicine. Stage 1 (initial encounter, like an emergency room scenario) included chief complaint, histories, vitals, and physical exam, without lab/imaging results. Stage 2 simulated the next clinical step, incorporating basic laboratory results and initial imaging studies typically available within a short timeframe (eg, complete blood count, metabolic panel, chest X-ray, and electrocardiogram). Stage 3 (CPSolvers cases only) added specialized lab tests and advanced imaging (excluding definitive tests), which generally take longer for results.

The allocation of specific clinical information to each stage, while not governed by a rigid, itemized checklist for every data point, was based on the collective clinical judgment of the authors. The goal was to create a realistic and logical progression of a diagnostic workup, ensuring that the “true” diagnosis was adequately supported by the cumulative information available at the final stage of each case.

To reduce data contamination risk and ensure valid performance evaluation, we took measures to minimize the chance that LLMs had prior knowledge of cases in this study. Common clinical cases were newly created by our internal medicine resident team members and are not publicly available, reducing their likelihood in LLM training data.

The complex cases in our study were inspired by real patient scenarios from publicly available CPSolvers morning rounds, reflecting realistic clinical complexity. However, to maintain originality and research relevance, the specific case presentations, including disclosed information and wording, were uniquely created by our team and are not directly copied from CPSolvers materials. This approach allowed us to capture authentic clinical complexity while generating novel scenarios unlikely to appear in LLM training datasets.

### Model selection

To address the gap in understanding which LLMs perform best in the medical domain, we selected multiple leading models from 3 major AI providers: Anthropic, OpenAI, and Google. This approach allows for directly comparing their capabilities within the same clinical scenarios. We documented specific version identifiers for each model, as AI companies frequently update their models and eventually deprecate older versions. A comprehensive list of models, their providers, and their specific versions is available in Table 1. For consistency, throughout the manuscript, we refer to models by their model names.

**Table 1.** Large language models used in the study.

Provider	Model name	Version identifier
Anthropic	Claude 3.5 Sonnet	claude-3-5-sonnet-20241022
Anthropic	Claude 3.5 Haiku	claude-3-5-haiku-20241022
Anthropic	Claude 3.7 Sonnet	claude-3-7-sonnet-20250219
Anthropic	Claude 3.7 Thinking	claude-3-7-sonnet-20250219_thinking
Anthropic	Claude 3 Opus	claude-3-opus-20240229
OpenAI	GPT-4o Latest	chatgpt-4o-latest
OpenAI	GPT-4o	gpt-4o-2024-11-20
OpenAI	GPT-4o Mini	gpt-4o-mini-2024-07-18
OpenAI	GPT-4	gpt-4-0613
OpenAI	GPT-3.5 Turbo	gpt-3.5-turbo-0125
OpenAI	O1 (High)	o1-2024-12-17_high
OpenAI	O1 (Medium)	o1-2024-12-17_medium
OpenAI	O1 Mini	o1-mini-2024-09-12
OpenAI	O1 Preview	o1-preview-2024-09-12
OpenAI	O3-mini (High)	o3-mini-2025-01-31_high
OpenAI	O3-mini (Medium)	o3-mini-2025-01-31_medium
Google	Gemini 1.5 Flash	gemini-1.5-flash-002
Google	Gemini 1.5 Flash 8B	gemini-1.5-flash-8b-001
Google	Gemini 1.5 Pro	gemini-1.5-pro-002
Google	Gemini 2.0 Flash	gemini-2.0-flash-001
Google	Gemini 2.0 Pro	gemini-2.0-pro-exp-02-05
Google	Gemini 2.0 Thinking	gemini-2.0-flash-thinking-exp-01-21

### Experimental procedure

An autonomous system was developed using Python programming to interact with the LLMs through their application programming interfaces (APIs). The system processed each version of the patient cases (stages 1-3) by sending the clinical information to the LLMs. For each case and stage, the LLMs were prompted to generate a differential diagnosis list, provide reasoning for each potential diagnosis, and identify a primary diagnosis based on the supplied information.

The LLM outputs were systematically collected and organized into a tabular format for analysis. The system documented both the proposed diagnoses and the models’ corresponding rationales. All LLM responses for each case are presented in Table S1.

### Performance evaluation

**Figure 1.**
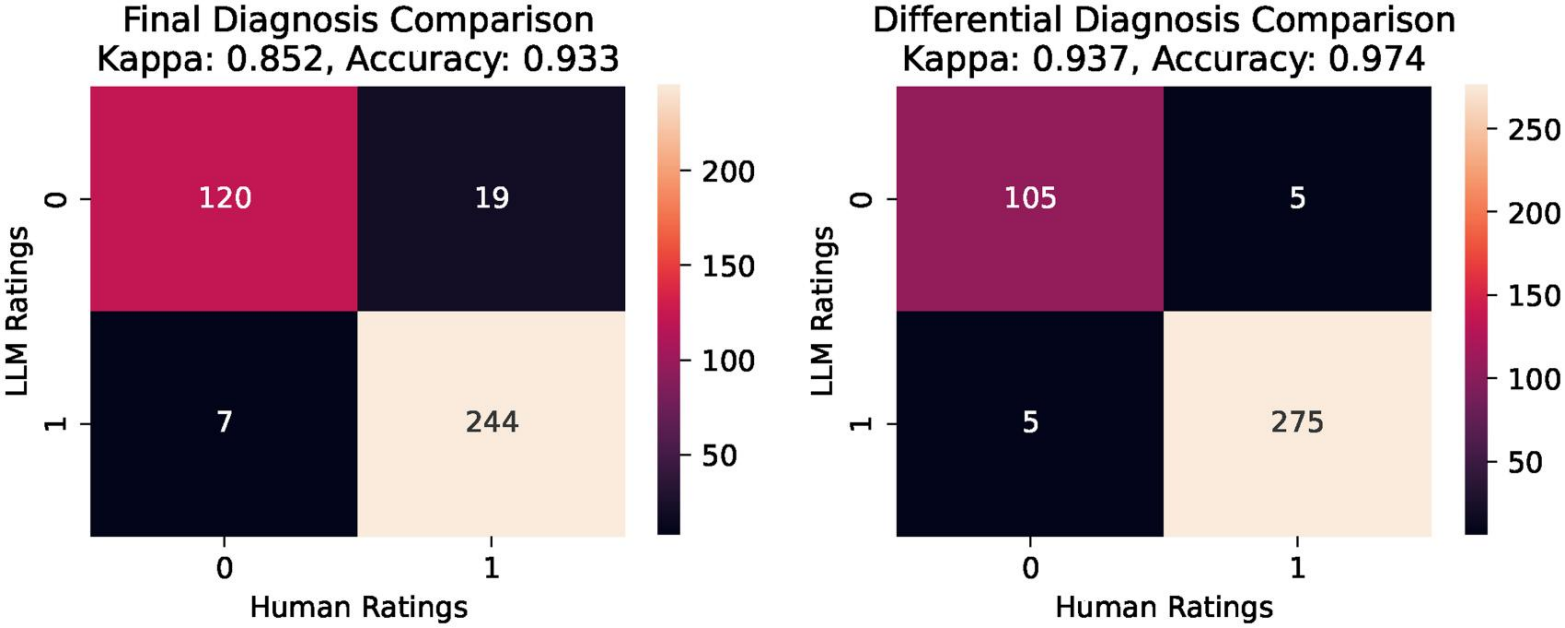
Interrater agreement analysis of LLM-based and human evaluations. Confusion matrices depict interrater agreement between large language model (LLM)-based evaluations and human evaluations across randomly selected cases (*n* = 390). Left panel: Final diagnosis comparison showing substantial agreement with Cohen’s Kappa (*κ*)=0.852 and accuracy = 93.3%. Right panel: Differential diagnosis comparison demonstrating near-perfect agreement with Cohen’s Kappa (*κ*)=0.937 and accuracy = 97.4%. These findings confirm the reliability and robustness of the LLM-based automated evaluation method employed in this study.

We evaluated diagnostic accuracy using a 2-tiered approach: automated LLM assessment and human validation. This novel method LLMs as a scalable evaluator, with expert human review for reliability. Automated assessment compared LLM outputs to predefined “true” diagnoses, evaluating final diagnosis accuracy and comprehensiveness of differential diagnoses. For each case, LLM outputs using predefined clinical criteria: 1 point was awarded for inclusion of the true diagnosis based on criteria encompassing exact matches, clinically related diagnoses (same pathophysiology and disease category) and 0 points otherwise. This enabled efficient evaluation across numerous cases. To validate automated scoring, we conducted interrater reliability testing on 390 randomly selected cases. Scores from LLM were compared to those of internal medicine residents, our human reference standard. Agreement, measured by Cohen’s Kappa (*κ*), was strong (lowest *κ* = 0.852), demonstrating high consistency between automated and human evaluation and supporting the method’s reliability (Figure 1).

### Top-*k* accuracy analysis

To assess the relative importance of primary vs differential diagnosis accuracy, we conducted a top-*k* accuracy analysis on a random subset (40 common clinical presentations and 50 complex CPSolvers cases) using representative models from our dataset. We modified the prompt to explicitly request a ranked differential diagnosis, instructing each model to provide at least 10 diagnoses ranked from most to least likely. Accuracy was measured at 3 thresholds: top-1 (*k*1), top-5 (*k*5), and top-10 (*k*10). A diagnosis was considered correct if the true diagnosis appeared within the specified top-*k* positions using the same criteria in the prior analysis. This allowed evaluation of how often models correctly identified the primary diagnosis vs including it within their broader differential.

### Software, tools, and prompts

The study used Python programming for core functionality, including API interactions with OpenAI, Anthropic, and Google’s LLMs, automated case processing, and evaluation systems. The autonomous system streamlined clinical case input, model interaction, and output collection through official API implementations. Visualization and figure generation were conducted in R using the ggplot2 package, enabling the creation of publication-quality graphics.

### Statistical analysis

Statistical analyses were performed using R version 4.4.1 and python 3.11. Diagnostic accuracy was calculated for each LLM at each stage of information disclosure as the mean proportion of correct responses. Standard errors were calculated for descriptive purposes and visualization.

Interrater reliability between the automated LLM-based scoring system and human expert ratings was assessed using Cohen’s Kappa on a subset of 390 randomly selected evaluations. Overall accuracy, sensitivity, and specificity of automated scoring relative to human ratings were also calculated. Data handling was done with pandas, and statistical metrics were computed using scikit-learn in Python.

Overall differences in diagnostic accuracy among models were assessed using the Chi-square test for both common and complex case sets at relevant stages. Following significant Chi-square results, pairwise comparisons with Chi-square were performed to identify specific differences between models. Table S2 shows the accuracy results and 95% CIs for all models when predicting final diagnoses and differential diagnoses in both common and complex cases.

For top-*k* accuracy analysis, we conducted 2 statistical comparisons: intermodel and intramodel. For intermodel comparisons, we tested overall accuracy differences among models under identical conditions (case type, stage, k-metric) using Chi-square tests. For intramodel comparisons, we assessed accuracy changes due to information stage (stage 1 vs stage 2, stage 2 vs stage 3, stage 1 vs stage 3) and k-metric threshold (k1 vs k5, k5 vs k10) within each model. To approximate comparisons of identical cases under varying conditions, we performed Chi-square tests on contingency tables of correct/incorrect responses per condition. For multiple comparisons within each model and case type. This allowed us to identify significant accuracy differences as information increased (stage progression) and as more differential diagnoses were considered (higher *k*-values).

### Ethical considerations

The study involved secondary analysis of deidentified patient cases. Appropriate consent was obtained from CPSolvers for the use of their publicly available cases. As no identifiable patient information was included and there was no direct patient interaction, the study was classified as exempt from Institutional Review Board (IRB) approval in accordance with institutional guidelines.

## Results

### LLMs demonstrate excellent diagnostic accuracy in common clinical presentations

**Figure 2.**
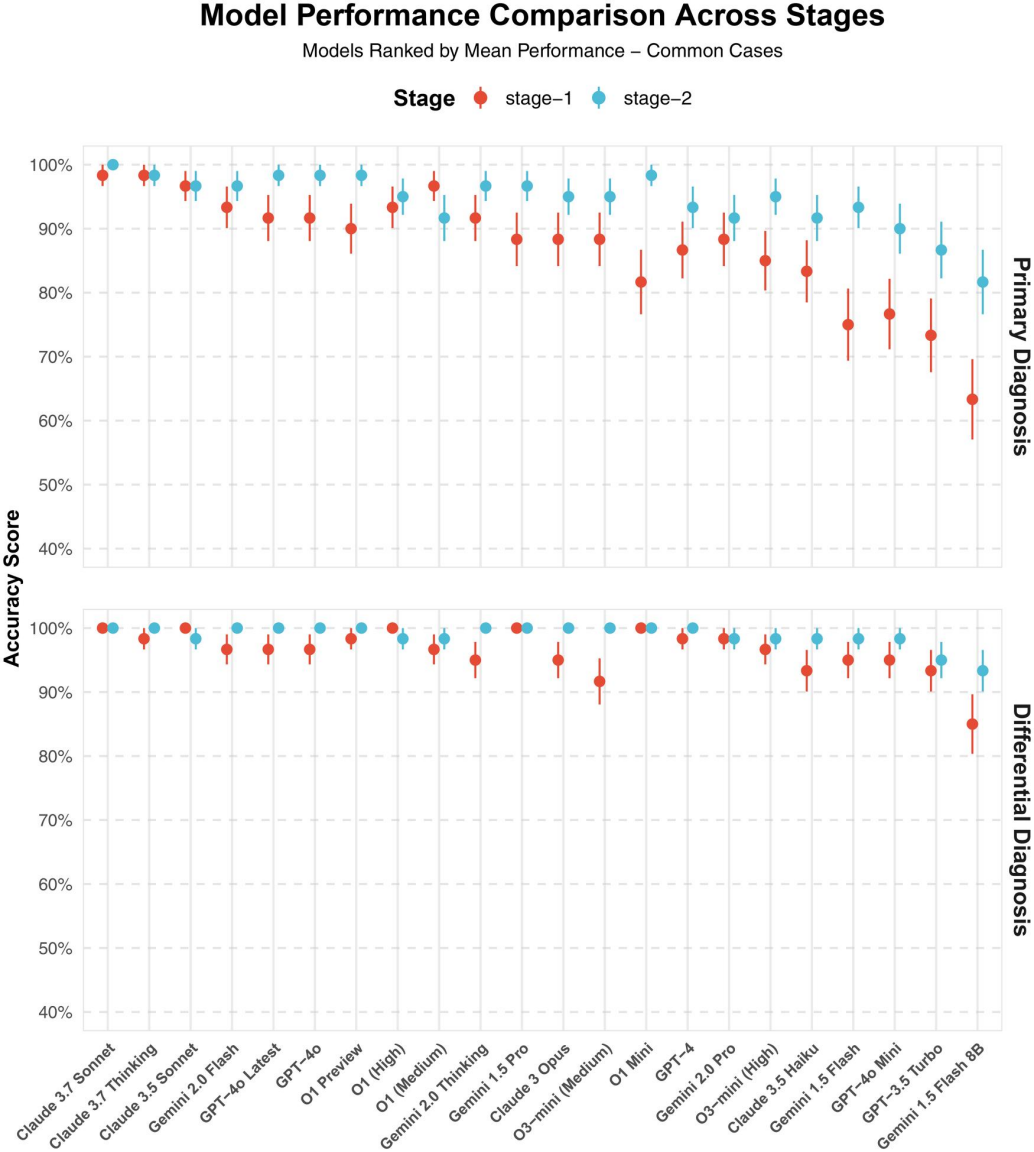
Model performance comparison across diagnostic stages for common clinical cases. The figure illustrates accuracy scores of various large language models (LLMs) for primary and differential diagnoses across 2 stages of clinical information disclosure: stage 1 (subjective information and physical examination only) and stage 2 (addition of basic laboratory and imaging data). Models are ranked by their mean performance across both stages. Upper panel: Primary diagnosis accuracy shows marked improvement from stage 1 to stage 2, with advanced models such as Claude 3.7 Sonnet and Claude 3.7 Thinking achieving near-perfect to perfect accuracy. Lower panel: Differential diagnosis accuracy remains consistently high across models, with multiple advanced models maintaining perfect accuracy. Error bars represent the SEM.

Our analysis evaluated the diagnostic capabilities of multiple LLMs across common clinical presentations in 2 distinct stages: stage 1 (subjective information and physical examination only) and stage 2 (addition of basic laboratory and imaging data). As illustrated in Figure 2, several advanced LLMs demonstrated exceptional accuracy in stage 1. Claude 3.7 variants (Sonnet and Thinking) achieved the highest primary diagnosis accuracy (98.3%), followed closely by Claude 3.5 Sonnet (96.7%). GPT-4o variants (including GPT-4o Latest) and Gemini 2.0 Flash also showed strong performances, with accuracies of approximately 91.7% and 93.3%, respectively. Smaller models, such as GPT-4o Mini and Claude 3.5 Haiku, performed well too, achieving accuracies ranging from 76.7% to 83.3%.

Stage 2, which included basic diagnostic data, further improved accuracy. Claude 3.7 models achieved perfect primary diagnosis accuracy (100%), while Gemini 2.0 Flash and GPT-4o Latest reached near-perfect performance (96.7%-98.3%). Base-level models like Gemini 1.5 Flash 8B and GPT-3.5 Turbo also significantly improved compared to stage 1, surpassing 80% accuracy. A Chi-square analysis indicated statistically significant overall differences between models (χ^2^ = 45.55, *P* = .0015); however, comprehensive pairwise comparisons revealed no significant differences among advanced models (eg, Claude 3.7 variants, GPT-4o variants, Gemini 2.0 Flash; all *P* > .05). Advanced models consistently demonstrated significant superiority compared to base-level models (eg, Gemini 1.5 Flash, GPT-3.5 Turbo; *P* < .05).

Differential diagnosis accuracy demonstrated even stronger and more consistent results across models. Claude 3.7 variants consistently demonstrated perfect (100%) differential diagnosis accuracy in both stages. Gemini 2.0 Flash, GPT-4o variants, and O1 series models consistently achieved accuracy above 96%. Notably, smaller models such as O1 Mini and Gemini 1.5 Pro also reached perfect differential diagnosis accuracy, highlighting that precision was not strictly dependent on model complexity or size.

### Differential diagnostic performance of LLMs in complex clinical cases

We evaluated the diagnostic performance of various LLMs across 104 challenging clinical cases from CPSolvers’ morning rounds, characterized by atypical presentations, rare conditions, and multisystem involvement. The evaluation followed a 3-stage diagnostic process with increasing informational complexity: initial clinical presentation (stage 1), addition of basic laboratory and imaging data (stage 2), and incorporation of advanced diagnostics such as MRI, endoscopy, and biopsy (stage 3).

**Figure 3.**
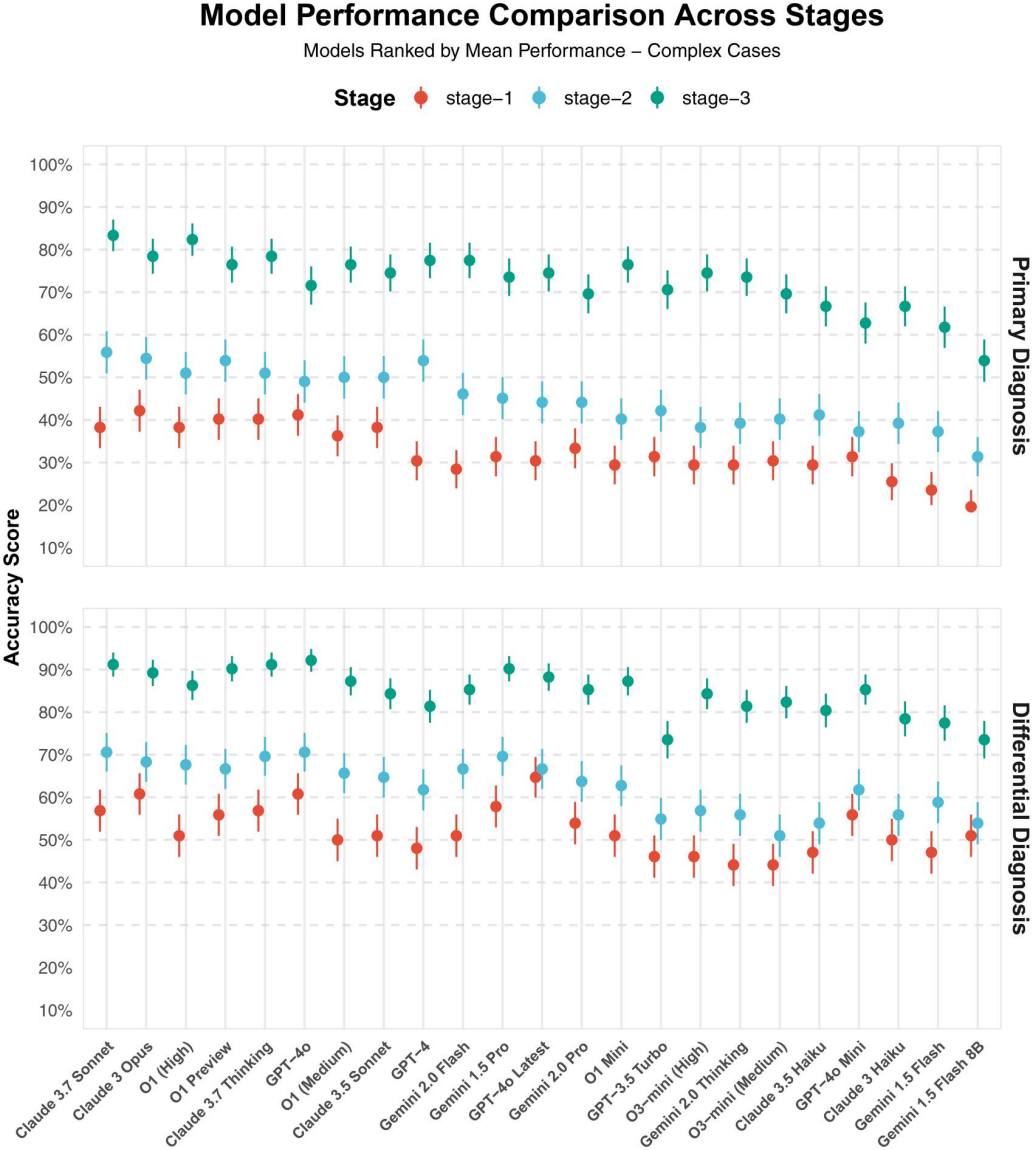
Model performance comparison across diagnostic stages for complex clinical cases. This figure displays the accuracy scores of various large language models (LLMs) for primary diagnosis (upper panel) and differential diagnosis (lower panel) across 3 stages of clinical information disclosure: stage 1 (initial presentation with subjective information and physical examination only), stage 2 (addition of basic laboratory and imaging data), and stage 3 (incorporation of advanced diagnostic studies). Models are ranked by their mean performance for primary diagnoses across all stages. The chart demonstrates a progressive improvement in diagnostic accuracy with increasing clinical information, with Claude 3.7 Sonnet achieving the highest primary diagnosis accuracy (83.3%) at stage 3, followed closely by O1 (High) at 82.4%. For differential diagnosis, GPT-4o led at stage 3 with 92.2% accuracy, with Claude 3.7 variants (91.2%) and Claude 3 Opus (89.2%) performing strongly as well. Error bars represent the SEM.

Diagnostic accuracy improved notably across all models with each additional stage of information (Figure 3). At stage 1, Claude 3 Opus achieved the highest primary diagnosis accuracy (42.2%), closely followed by GPT-4o (41.2%) and Claude 3.7 Sonnet (Thinking) and O1 Preview (each 40.2%). Stage 2 showed substantial improvements, with Claude 3.7 Sonnet (55.9%) slightly outperforming Claude 3 Opus (54.5%), GPT-4, and O1 Preview (each 53.9%).

At stage 3, significant performance enhancements were observed, with Claude 3.7 Sonnet achieving the highest primary diagnosis accuracy (83.3%), closely followed by O1 (High) at 82.4%, Claude 3 Opus at 78.4%, and GPT-4 at 77.5%. Overall Chi-square analysis indicated significant differences among all tested models (χ^2^=57.77, *P* < .001). Comprehensive pairwise comparisons revealed that while many leading models, such as Claude 3.7 Sonnet, O1 (High), Claude 3 Opus, and GPT-4, showed no statistically significant differences among each other (all *P* > .05), advanced models consistently demonstrated statistically significant superiority compared to smaller or less advanced models (eg, Gemini 1.5 Flash, Gemini 1.5 Flash 8b, O3 Mini, and O1 Mini; all *P* < .05). In generating differential diagnoses, GPT-4o led at stage 3 with a differential diagnosis accuracy of 92.2%, followed closely by Claude 3.7 Sonnet variants (91.2%) and Claude 3 Opus (89.2%).

### Evaluating top-*k* differential diagnosis performance of LLMs

**Figure 4.**
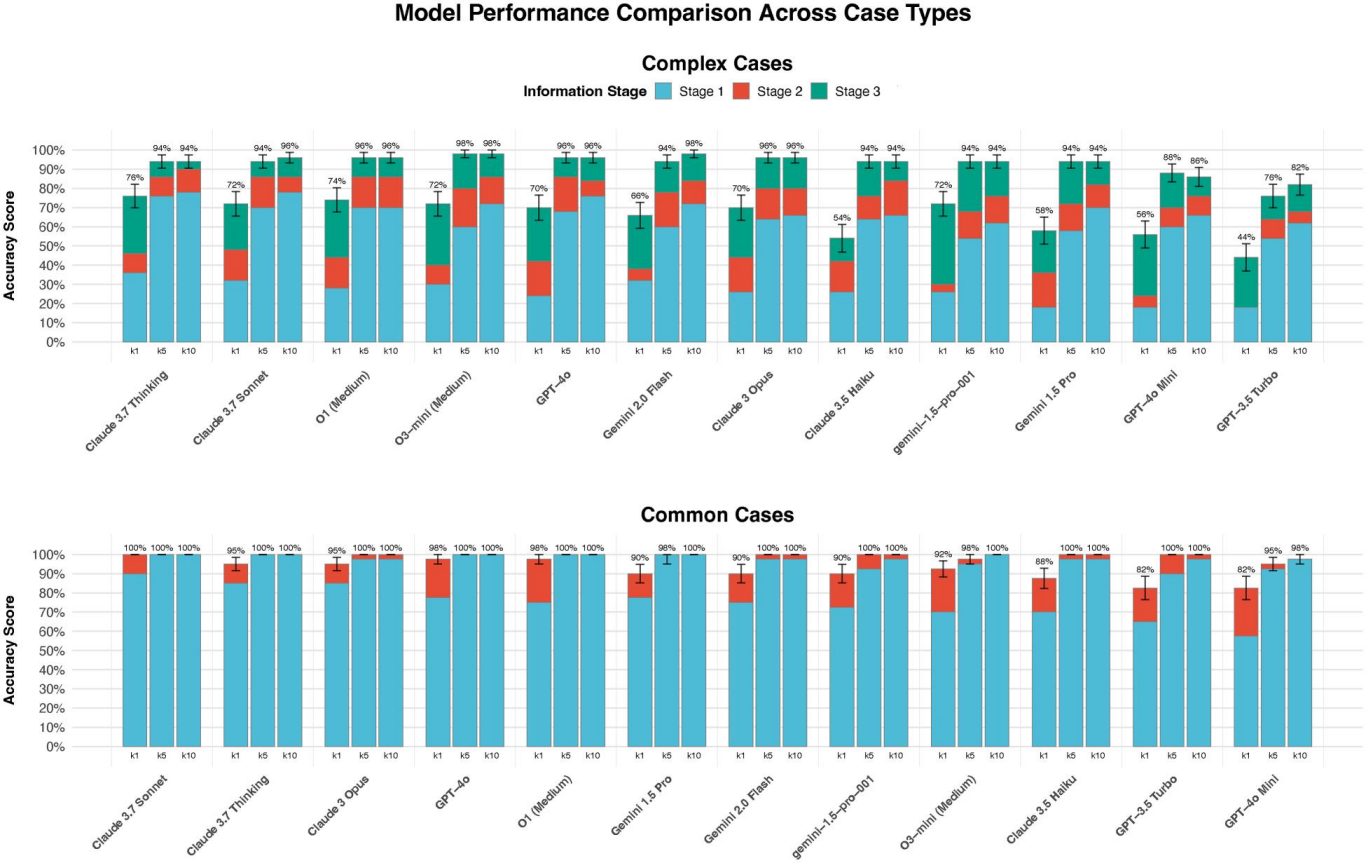
Model performance comparison for top-*k* accuracy by case type and information stage. This figure shows top-*k* diagnostic accuracy (*k* = 1, *k* = 5, *k* = 10) of various LLMs across clinical information stages, comparing complex cases (upper panel) and common cases (lower panel). The k = 1 accuracy typically represents primary diagnosis accuracy, while k = 5 and k = 10 indicate correct diagnosis inclusion within broader differentials. Upper panel (Complex Cases): Performance is shown across 3 clinical information stages: stage 1 (initial subjective information and physical exam only), stage 2 (addition of basic lab and imaging data), and stage 3 (advanced diagnostic studies). Lower panel (Common Cases): Performance is shown across 2 information stages: stage 1 (subjective information and physical exam only) and stage 2 (addition of basic lab and imaging data). Error bars indicate SEM.

To assess LLM diagnostic reasoning beyond single-best accuracy, we performed a top-*k* analysis on 40 common and 50 complex cases using representative models (Figure 4). Models were prompted for a ranked list of ≥10 differential diagnoses. Accuracy was measured by the correct diagnosis’s presence in the top-1 (*k*1), top-5 (*k*5), or top-10 (*k*10) predictions at each information stage (Figure 4).

For common cases (Figure 4, lower panel), all models achieved high accuracy. At stage 2 (k1), Claude 3.7 Sonnet reached 100%, followed by GPT-4o (97.5%), O1 (Medium) (97.5%), Claude 3.7 Opus (95.0%), and Claude 3.7 Thinking (95.0%). Most models hit 100% at k5/k10 by stage 2. Chi-square tests showed no significant intermodel differences, indicating consistently strong performance.

Complex cases revealed greater intermodel differences, especially at k1, underscoring the value of k5/k10 analysis. By stage 3 (with advanced diagnostic information), k1 accuracy substantially improved. Top k1 performers at stage 3 were Claude 3.7 Thinking (76.0%), O1 (Medium) (74.0%), and Claude 3.7 Sonnet, O3-mini (Medium), gemini-1.5-pro-001 (all 72.0%). Chi-square analysis showed significant k1 accuracy differences among models at stage 3 (*P* = .012).

In complex cases, most models showed significant k1 accuracy improvements from stage 1 to stage 3 (eg, Claude 3.7 Thinking *P* = .0012; O1 [Medium] *P* = .0001; Claude 3.7 Sonnet *P* = .0013). Stage 2 to stage 3 improvements were also significant for models like Claude 3.7 Thinking (*P* = .0369), O1 (Medium) (*P* = .0398), and O3-mini (Medium) (*P* = .0226).

Comparing k-metrics for complex cases, extending from k1 to k5 significantly improved accuracy at all stages (all *P* < .05, Bonferroni corrected). However, k5-k10 showed no significant improvement for any model or stage.

### Qualitative analysis of key cases highlights LLM reasoning

Qualitative review of selected clinical scenarios showed variability in LLM reasoning, revealing both sophisticated clinical insights and diagnostic oversights. Advanced models often demonstrated strong reasoning. For example, in Case 1397 (81-year-old suburban male with altered mental status and high fever), models like Claude 3.7 Sonnet and OpenAI O1 (high reasoning) accurately identified West Nile virus at stage 1. Their reasoning effectively incorporated epidemiological details—suburban residence increasing mosquito exposure risk—alongside advanced age and neurological symptoms, avoiding generic diagnoses. In contrast, O3 Mini considered encephalitis but failed to factor in suburban living, limiting its ability to prioritize West Nile virus.

Demographic data also challenged certain models. In case 1355, involving a young female with symptoms suggestive of NMDA autoimmune encephalitis—strongly associated with this demographic—advanced models (Claude 3.7 Sonnet, Claude 3.5 Sonnet) correctly identified NMDA encephalitis, explicitly citing age and gender. Other models, such as O3 Mini (high mode), missed NMDA encephalitis and omitted demographic considerations. Similarly, Claude 3.5 Haiku proposed rabies (possible but less likely), inadequately weighing demographic factors and characteristic psychiatric features with diffuse muscle stiffness. These examples illustrate how neglecting demographic risk factors can mislead certain LLMs.

Additionally, models struggled with appropriate weighting of diagnostic probabilities. In case 1359 (rhabdomyolysis from cocaine use), Claude 3.7 Sonnet correctly identified rhabdomyolysis by emphasizing cocaine exposure and kidney injury (anuria). In contrast, O3 Mini and O1 suggested cocaine-induced spinal cord infarction—a much rarer complication—attributing urinary retention to less likely mechanisms (autonomic involvement from spinal injury) rather than the more common rhabdomyolysis-induced acute kidney injury. This highlights difficulties certain LLMs have in aligning diagnoses with clinical likelihood.

Lastly, performance varied even with common diagnoses presented atypically. Advanced models often correctly prioritized COVID-19 despite nonspecific symptoms (case 25) or initial negative rapid tests (case 26), demonstrating awareness of false-negative rates. However, this nuanced understanding was not universal across tested models.

## Discussion

This study offers a thorough and systematic comparison of leading LLMs developed by major AI providers, within the context of clinical diagnosis. Unlike previous studies, which have generally examined a smaller group of LLMs or used simpler question formats,^11^^,^^16–18^ our research directly tested multiple advanced models against each other. We used complex clinical cases drawn from CPSolvers morning rounds, as well as common clinical presentations adjusted to include subtle diagnostic variations. This approach was carefully designed to reflect the complexity and nuance of real-world diagnostic scenarios faced by clinicians.

Our findings reveal several key insights. Firstly, most advanced LLMs demonstrated impressive diagnostic accuracy in typical clinical scenarios. This suggests that while routine cases may not fully differentiate the capabilities of these models, they nonetheless possess a strong baseline competence in frequently encountered medical presentations. Interestingly, even smaller models performed comparably well in these common cases, showing no statistically significant difference compared to larger models and suggesting potential cost-effective deployment options for routine diagnostic support. However, including complex real-world cases from CPSolvers was important for clearly revealing performance differences between models. These complex cases highlight how advanced large language models, like Anthropic’s Claude 3.7 Sonnet and OpenAI’s o-series reasoning models, have improved diagnostic capabilities. This performance difference becomes clear when looking at the progression of LLMs. GPT-3.5 was groundbreaking when first released but now sits among the lowest performers in our study. In contrast, Claude 3.7 Sonnet, introduced just a few months ago, shows outstanding diagnostic accuracy.

A key strength of our study is the staged approach to information disclosure. By gradually presenting clinical data across 3 stages, we closely mirrored the step-by-step process of real-world diagnostic practice. This unique method allowed us to observe how advanced LLMs maintain high accuracy and improve diagnostic precision as additional clinical information becomes available, even in challenging cases with subtle or unusual presentations. Our intramodel analysis confirmed that for complex cases, adding stage 1 data to stage 3 significantly improved top-1, top-5, and often top-10 accuracy across nearly all tested models. Additionally, several leading models showed notable top-1 accuracy gains even from stage 2 to stage 3, indicating advanced diagnostic data further refines their primary diagnosis. These findings highlight the potential of LLMs to assist clinicians, particularly when facing difficult diagnostic cases.

Our top-*k* analysis revealed another key insight. Considering the model’s top 5 differential diagnoses, rather than only the primary diagnosis, markedly improves diagnostic accuracy across nearly all models, stages, and case types. This finding has clear clinical implications, suggesting LLM-based diagnostic tools should provide multiple differential options rather than a single diagnosis. Interestingly, expanding the differential from 5 to 10 did not yield significant additional benefit in any tested scenario, indicating a practical upper limit for clinicians using these tools in practice.

Additionally, we introduced and validated a new LLM-based evaluation method. By using an LLM as an automated rater, we carefully verified its reliability through strong agreement with human expert assessments (*κ* = 0.85). This allowed us to develop an efficient and scalable approach to measure the diagnostic performance of LLMs. Thanks to this innovation, we were able to reliably analyze a large dataset of over 9000 data points. Evaluating something of this scale would have been nearly impossible with manual rating alone. Our method thus provides a valuable resource for future research, especially when conducting large-scale comparisons of AI models in medicine.

In contrast to a prior meta-analysis by Waldock et al,^19^ which reported a modest 51% accuracy for AI in general medical examinations, our findings demonstrate a significantly more advanced reality. That meta-analysis mainly looked at earlier generations of LLMs, so it did not fully represent the capabilities of today’s rapidly improving models. In fact, newer LLMs such as GPT-4o now achieve near-perfect scores on medical licensing exams.^20^^,^^21^ Our own comparisons clearly show this advancement: newer models consistently performed better than earlier GPT versions. This clear distinction highlights why regular evaluations using newer models are essential. Such up-to-date assessments help us accurately grasp AI’s rapidly evolving potential in medicine.

Although recent advancements are promising and state-of-the-art LLMs have demonstrated strong diagnostic capabilities, a key challenge remains in translating this accuracy into improvements for clinicians making diagnoses in real-world medical practice.^11^^,^^22^ This situation, referred to as the “AI paradox,” highlights an urgent need to establish structured guidelines for integrating LLM-based clinical decision support tools effectively and safely. Future research should focus on addressing practical implementation issues. Researchers should examine how LLMs can incorporate important contextual factors, such as patient demographics, geographic location, seasonal changes in disease occurrence, and local epidemiological outbreaks. Addressing these details will help ensure diagnostic suggestions are relevant and prevent inappropriate recommendations. Additionally, studies must evaluate how LLM-based platforms can most effectively obtain and integrate this type of contextual input from clinicians, further improving diagnostic accuracy across diverse clinical situations.

Our study provides valuable insights into the comparative diagnostic capabilities of LLMs within a staged clinical reasoning framework, though certain limitations warrant consideration. The clinical scenarios used are solely text-based and primarily focused on internal medicine presentations, limiting generalizability to other clinical specialties and multimodal diagnostic contexts. Although we developed common cases internally and substantially adapted complex cases from CPSolvers to mitigate data contamination (as detailed in “Methods”), the possibility of conceptual overlap with existing LLM training data cannot be completely excluded. Additionally, while our automated LLM-based scoring approach showed high concordance with a human rater, it relies on binary diagnostic accuracy and does not account for complex decision-making or nuanced aspects of clinical reasoning, such as diagnostic confidence and rationale quality. Additionally, our study did not examine how patient factors (eg, age, gender, race, comorbidities) influence LLM reasoning and diagnostic outputs. Understanding these factors is crucial for assessing the equity and generalizability of AI systems across diverse patient populations; therefore, omitting them is a limitation of our work. Furthermore, although validated against human raters, using an LLM as an evaluator carries inherent risks of circularity or unforeseen biases, a challenge in large-scale AI assessment that future research should address. Beyond diagnostic accuracy, it is important that the tool is accessible and easy to use across different clinical settings. Future research must explore the practical integration of LLMs in resource-constrained environments and assess their utility not only for primary diagnosis generation but also for supporting diagnostic review and clinical judgment validation across different levels of clinician expertise. Finally, we must proactively address the potential impact on medical education. Concerns regarding over-reliance on AI-generated diagnoses potentially hindering the development of clinical reasoning skills in trainees necessitate careful consideration. Medical educators must develop pedagogical strategies to effectively integrate these powerful diagnostic tools into curricula in a manner that complements, rather than replaces, the development of essential diagnostic competencies.

In conclusion, our comprehensive evaluation confirms the significant potential of state-of-the-art LLMs to enhance diagnostic accuracy in both common and complex cases. The notable improvements observed across stages of information disclosure, along with the clear statistical advantage of considering multiple differential diagnoses over a single primary diagnosis, offer actionable guidance for clinical practice. However, realizing this potential requires bridging the gap between model capability and clinical integration. Critically, given the rapid pace of LLM advancements, ongoing evaluation is vital. Future efforts must prioritize robust implementation frameworks, address contextual relevance and equitable access, thoughtfully integrate LLMs into medical education, and establish continuous testing mechanisms to keep pace with rapidly evolving models. This iterative approach is essential to maximize the benefits of AI in medicine and ensure optimal patient care in this dynamic field.

## Supplementary Material

ooaf055_Supplementary_Data

## Data Availability

All data generated or analyzed during this study are included in this published article and in the Supplementary Material. Additional data can be requested from the corresponding author upon reasonable request.
